# The Herbicide Atrazine Potentiates Angiotensin II-Induced Aldosterone Synthesis and Release From Adrenal Cells

**DOI:** 10.3389/fendo.2021.697505

**Published:** 2021-07-14

**Authors:** Arthur D. Zimmerman, Laci Mackay, Robert J. Kemppainen, Melaney A. Jones, Casey C. Read, Dean Schwartz, Chad D. Foradori

**Affiliations:** Department of Anatomy, Physiology and Pharmacology, College of Veterinary Medicine, Auburn University, Auburn, AL, United States

**Keywords:** atrazine, herbicide, adrenal, aldosterone, angiotensin II, H295R

## Abstract

Atrazine is one of the most commonly used pre-emergence and early post-emergence herbicides in the world. We have shown previously that atrazine does not directly stimulate the pituitary or adrenal to trigger hormone release but acts centrally to activate a stress-like activation of the hypothalamic-pituitary-adrenal axis. In doing so, atrazine treatment has been shown to cause adrenal morphology changes characteristic of repeated stress. In this study, adrenals from atrazine treated and stressed animals were directly compared after 4 days of atrazine treatment or restraint stress. Both atrazine and stressed animals displayed reduced adrenocortical zona glomerulosa thickness and aldosterone synthase (CYP11B2) expression, indicative of repeated adrenal stimulation by adrenocorticotropic hormone. To determine if reduced CYP11B2 expression resulted in attenuated aldosterone synthesis, stressed and atrazine treated animals were challenged with angiotensin II (Ang II). As predicted, stressed animals produced less aldosterone compared to control animals when stimulated. However, atrazine treated animals had higher circulating aldosterone concentrations compared to both stressed and control groups. Ang II-induced aldosterone release was also potentiated in atrazine pretreated human adrenocortical carcinoma cells (H295R). Atrazine pretreated did not alter the expression of the rate limiting steroidogenic StAR protein or angiotensin II receptor 1. Atrazine treated animals also presented with higher basal blood pressure than vehicle treated control animals suggesting sustained elevations in circulating aldosterone levels. Our results demonstrate that treatment with the widely used herbicide, atrazine, directly increases stimulated production of aldosterone in adrenocortical cells independent of expression changes to rate limiting steroidogenic enzymes.

## Introduction

Atrazine (2-chloro-4-ethylamino-6-isopropylamino-s-triazine) is one of the most commonly used herbicides worldwide (1). Atrazine is used to control annual grasses and broadleaf weeds in agricultural crops such as corn, sugarcane, and sorghum, as well as on urban golf courses with an estimated annual application of 80 million pounds (2). Atrazine’s widespread use and relatively long half-life makes it the most commonly detected herbicide in water sources (3, 4). In plants, atrazine inhibits photosynthesis in plants by binding to the D1 protein located in the thylakoid membrane of chloroplasts, thereby blocking electron transport necessary for photosynthesis (5, 6).

In rats, the effects of atrazine exposure on the hypothalamic-pituitary-gonadal (HPG) axis have been well documented. In particular, we have shown that atrazine will inhibit both pulsatile and preovulatory surge release of gonadotropin releasing hormone (GnRH) and the subsequent release of luteinizing hormone (LH) from the pituitary (7–9). The mechanism by which atrazine inhibits GnRH and LH release is not fully understood. It is possible that the inhibitory effects of atrazine and its chlorometabolites on gonadotropins (8–10) is partially due to the interaction between the chlorotriazine and the hypothalamic-pituitary-adrenal (HPA) axis. All evidence suggests, atrazine acts centrally in the brain to induce this response. Central infusion of atrazine or molar equivalent doses of its primary metabolites, results in a rapid rise in stress hormones. Atrazine does not induce adrenocorticotropic hormone (ACTH) release in pituitary cell cultures. Additionally, the blockade of corticotropin-releasing hormone (CRH) receptors inhibits atrazine-induced elevation in corticosterone *in vivo*. This suggests that atrazine does not directly stimulate ACTH release from the pituitary, but requires CRH receptor activation, presumably from hypothalamic derived CRH. Likewise, the concurrent rise in plasma ACTH and corticosterone concentrations after atrazine treatment, and the lack of atrazine-induced corticosterone in animals under dexamethasone suppression or in animals without a pituitary, does not support a direct action of atrazine on adrenal gland steroidogenesis. However, we have termed atrazine’s effects on the HPA axis as “stress-like” since, although CRH receptor activation is required for atrazine-induced ACTH release, CRH cells of the hypothalamic paraventricular nucleus fail to display markers of activation after atrazine treatment (cFos and P-CREB) (11). Likewise, animals do not perceive atrazine as a stressor. Animals do not respond to atrazine treatment with hyperthermia or display avoidance behavior common in stressed animals (12).

Through our previous investigation, the zona glomerulosa (zG) of the adrenal cortex was found to be reduced in thickness after 4 days of atrazine treatment (11). As mentioned, atrazine treatment results in elevated concentrations in circulating ACTH similar to those found in restraint stressed animals (11, 13). ACTH treated or restraint stressed rats have similar reductions in zG thickness to atrazine treated animals (14, 15). We hypothesized that the changes in adrenal morphology induced by atrazine treatment would mimic those in restraint stressed animals (14). In addition, we investigated the ability of atrazine or stressed animals to mobilize aldosterone production in response to angiotensin II stimulation and possible effects on blood pressure. We hypothesized that rats exposed to repeated (4 days) atrazine treatment or stress (restraint) would display lower aldosterone plasma concentrations compared to control animals. While our findings do not support this hypothesis, it identified novel and profound effects of exposure to the ubiquitous herbicide, atrazine, on aldosterone synthesis and release both *in vitro* and *in vivo*.

## Methods

### Animals

All animal surgeries and experimental protocols were approved by the Animal Care and Use Committee of Auburn University (2015-2729) and were carried out in accordance with National Institutes of Health and Association for Assessment and Accreditation of Laboratory Animal Care guidelines. Young adult female Sprague Dawley rats (age 60 to 90 days) were obtained from Charles River Laboratories (Wilmington, MA). Animals were single- or double-housed and maintained on a 10-hour dark:14-hour light photoperiod (lights on at 0700 hours) with *ad libitum* access to food and water. The number of signal housed animals were distributed equally across treatment groups and the basal stress hormones from these animals were not statically different to pair-housed animals. All treatments were given within 2 hours of lights on. All blood sampling was completed within 5 hours of lights on. After ≥4 days to acclimate to the vivarium, all animals were bilaterally ovariectomized under 4% isoflurane followed by carprofen (Zoetis, Kalamazoo, MI) 2.2 mg/kg subcutaneously every 24 hours for 72 hours. Animals were allowed 7 to 10 days to recover before additional procedures were conducted. Ovarectomized-female rats were used in the current experiments to be consistent with our previous findings of atrazine-induced reduction in zG morphology (11). To reduce nonspecific stress associated with handling and gavage, animals used for all experiments were habituated for 4 to 7 days before treatment was initiated. A total of 135 animals were used in the described experiments.

### Test Substances

For animal studies, atrazine (45330; Sigma-Aldrich, Burlington, MA; certified, 98.8% pure) was prepared weekly as a suspension in 1% carboxymethylcellulose (CMC) sodium salt (9004-32-4; Alfa Aesar, Haverhill, MA) and deionized water. Suspensions were stored refrigerated (at 2°C to 8°C) until use. For cell culture studies, atrazine was dissolved in Dimethyl sulfoxide (DMSO; Sigma-Aldrich; 276855) and stored at -20°C until diluted into media. The *in vivo* dose and *in vitro* concentration selections were predicated on previous reports from our laboratories and others that reported repeatable and robust elevations in stress hormones after oral gavage of atrazine. The *in vivo* no effect level for the atrazine-induced activation of the HPA axis is 5 mg/kg in male rats (13), and the lowest observed effect level reported for female rats is 12.5 mg/kg (16). However, to characterize the effects of atrazine on adrenal morphology and activation, doses at the higher end of the known effective range to elicit a rise in stress hormone concentrations were used (11).

### Experiment 1: Effects of Atrazine or Restraint Stress Treatment on Adrenal Morphology

Animals were administered atrazine [100 mg/kg of body weight (BW)] in CMC by gavage once daily, CMC alone, or restraint stressed for 30 minutes in Plas-Labs Scientific restraint stress (Lansing, MI; 553-BSRR) for 4 consecutive days. Previous reports involving repeated stress and changes to adrenal morphology used 30 mins or 2 hours of daily restraint stress (14, 15). In the present studies, 30 mins of restraint stress was used because the hormone response (amplitude and duration of ACTH and corticosterone) to 30 mins of restraint is similar to that of atrazine (100mg/kg) treatment (11, 13). Animals were weighed daily. After completion of the last treatment, animals were euthanized by carbon dioxide inhalation, and the right adrenal gland was collected, weighed, and placed in 10% neutral-buffered formalin for immunohistochemical processing. Eight animals were used per treatment group.

### Experiment 2: Effects of Atrazine or Restraint Stress on Angiotensin II Induced Aldosterone Release

Animals were administered atrazine (100 mg/kg BW) in CMC by gavage once daily, CMC alone, or restraint stressed for 30 minutes in Plas-Labs Scientific restraint stress (Lansing, MI; 553-BSRR) for 4 consecutive days. On the 4^th^ day, but before the final treatment, animals were injected with dexamethasone (100 µg/kg in safflower oil, subcutaneously; 50-02-2; Sigma-Aldrich, St. Louis, MO). Atrazine and restraint stress have been shown to induce a rise in blood ACTH concentrations. Dexamethasone pretreatment was given on the final day of treatment to suppress ACTH release, to avoid ACTH-induced rise in aldosterone concentrations. 90 mins after dexamethasone, the gavage or restraint stress was administered. 45 minutes post treatment, animals were anesthetized with 4% isoflurane and an intra-atrial cannula was inserted for blood sampling, as described previously (17). Heparin saline (10 U) was injected *via* the jugular catheter. Approximately 60 min after final treatment, a baseline blood sample (50 µL) was taken and placed in heparin- and EDTA-treated tube on ice. Following the baseline sample, animals were treated with angiotensin II (Ang II, 5 µg/kg of BW; 4474-91-3; Alfa Aesar, Ward Hill, MA). From baseline sample (timepoint 0 min), samples were taken every 10 min for 40 min. Samples were spun at 1500 rpm for 15 minutes at 4°C. Plasma was frozen at −20°C until assayed for aldosterone. Seven animals were used per treatment group.

### Experiment 3: Effects of Atrazine Treatment Duration on Angiotensin II-Induced Aldosterone Release

Animals were administered atrazine in CMC (100 mg/kg BW), or CMC only for 1, 2, 3, or 4 consecutive days. On the final day of treatment animals were injected with vehicle (safflower oil, subcutaneously) or dexamethasone (100 µg/kg in safflower oil, subcutaneously; 50-02-2; Sigma-Aldrich, St. Louis, MO) and returned to their home cage. After 90 mins, the animals were anesthetized with 4% isoflurane. An intra-atrial cannula was inserted for blood sampling. Heparin saline (10 U) was injected *via* the jugular catheter. Forty-five minutes later a baseline blood sample (50 µL) was removed and placed in heparin- and EDTA-treated tube on ice. Following the baseline sample, animals were treated with angiotensin II (Ang II, 5 µg/kg of BW; 4474-91-3; Alfa Aesar, Ward Hill, MA). From baseline sample (timepoint 0 min), samples were taken every 10 min for 40 min. Samples were spun at 1500 rpm for 15 minutes at 4°C. Plasma was frozen at −20°C until assayed for aldosterone. Eight animals were used per treatment group.

### Experiment 4: Effects of Atrazine Treatment on Aldosterone Production in Human Adrenocortical Cells

NCl-H295R (H295R) cells were purchased through American Type Culture collection (ATCC CRL-2128; Manassas, VA). H295R cells were grown at 37°C and 5% CO_2_ in DMEM/F12 medium (Gibco Invitrogen, Carlsbad, CA) containing 2.5% FBS (HyClone, South Logan, UT), 2 mM L-glutamine (BioWhittaker Cambrex, East Rutherford, NJ), 1% ABAM (A5955, Sigma-Aldrich) and 1% ITS+ Premix Universal Culture Supplement (354352, Corning, Cleveland, TN). For analysis of response to atrazine and Ang II, cells were sub-cultured onto 12-well dishes at a density of 1.5 × 10^5^ cells/well.

#### Experiment 4a

One day before the experiment, cells were changed to low-serum experimental medium (1% FBS). After 16 hours, cells were changed to low-serum media containing 0.1, 1.0, or 10 µM of atrazine or the DMSO vehicle (final concentration <0.1%). After 24 hours of pretreatment, cultures were treated with 0.1 nM Ang II in fresh low-serum experimental medium containing the same atrazine or vehicle treatments. Post 24 hours of Ang II treatment, media was collected for aldosterone measurement and cells were collected for protein determination. Three biological replicates across 2-3 passages were used per treatment.

#### Experiment 4b

One day before the experiment, cells were changed to low-serum experimental medium (1% FBS). After 16 hours, cells were changed to media containing 10 µM of atrazine or the DMSO vehicle (final concentration <0.1%). After 1, 3, 6, 24, or 48 hours, cultures were treated with 0.1 nM Ang II in fresh low-serum experimental medium not containing atrazine. Post 24 hours of Ang II treatment, media was collected for aldosterone measurement and cells were collected for protein determination. Three biological replicates across 2-3 passages replicates were used per treatment.

### Experiment 5. Effects of Atrazine Treatment on Blood Pressure

Animals were administered atrazine in CMC (100 mg/kg BW), or CMC only for 4 consecutive days. Animals were weighed daily. On the final day of treatment, rats were maintained under 1.5% isoflurane in 3 liters/min oxygen throughout experiment. Carotid artery was cannulated for arterial pressure measurement, electrocardiographic wires attached to record Lead II ECG and the jugular vein was cannulated for fluid and drug infusion. Angiotensin II was infused at a rate of 0.1 ml/min at doses of 10, 30, 100, 300, 1000 ng/kg/min for 5 min or until arterial pressure stabilized. Phosphate buffered saline was then infused until arterial pressure returned to baseline before next infusion of angiotensin II. At the completion of the experiment, isoflurane was increased to 5% and heart and kidney removed and frozen in liquid nitrogen. Baseline values represent values before angiotensin II infusion. Increases in pressure represent the maximum increase in pressure in response to angiotensin II infusion. Eight animals were used per treatment group.

### Experiment 6. Effects of Atrazine Treatment on Select Gene Expression and Blood Electrolyte Levels

Animals were administered atrazine in CMC (100 mg/kg BW), or CMC only for 4 consecutive days. On the final day of treatment, animals were euthanized by cervical dislocation. Within 2 mins, blood samples were collected and analyzed with the iSTAT Portable Clinical Analyzer using the iSTAT CG8+ cartridge (03M86-01, iSTAT, Princeton, NJ). The iSTAT has previously been validated for rodent use (18, 19). Heart, kidney, and left adrenal tissue were collected and kept at -80°C until gene analysis. Five animals were used per treatment group.

### Immunohistochemistry

To determine the changes to adrenal morphology after repeated atrazine treatment, right adrenal glands were embedded in paraffin and 6-µm sections were placed on HistoBond slides. Slides were warmed at 55°C for 45 minutes, deparaffinized and rehydrated though xylene and graded ethanol. Sections were then antigen-unmasked by boiling in Tris buffer (10 mM Tris, 1 mM EDTA) at pH 9.0 for 15 minutes. Slides were washed in PBS (pH 7.2) and then placed in blocking solution (1% BSA in PBS) for 20 minutes. Slides were incubated in hybridization chambers overnight in a primary antibody cocktail in blocking solution [1:100 mouse anti–cytochrome P450 aldosterone synthase (Millipore, Billerica, MA; MAB6021; RRID: AB_95224) and 1:500 goat anti–11*β*-hydroxylase (Santa Cruz Biotechnology, Dallas, TX; sc-47652, RRID: AB_2088384)]. After incubation in primary antibody, slides were washed three times in PBS and then incubated in a fluorescent secondary antibody cocktail for 1 hour [1:250 Alexa Fluor 488 donkey–anti-mouse (Invitrogen; A21202) and 1:250 Alexa Fluor 594 donkey–anti-goat (A11058)]. After incubation in secondary antibody, slides were rinsed in phosphate buffer (pH 7.2) and then coverslipped with hardset mounting media with 4′,6-diamidino-2-phenylindole counterstain (Vector Laboratories, Inc., Burlingame, CA; H-1500). Slides were then imaged at ×20 magnification with a Perkin Elmer Cri-Nuance fluorescent multispectral imaging camera (Model FX; Perkin Elmer, Waltham, MA) in conjunction with a Zeiss Axioskop microscope (Carl Zeiss Microscopy, LLC, Thornwood, NY). With tunable filters matched to the bandwidths of molecular markers, the camera acquired spectral information from each sample, resulting in component images. The various signals from multiple labels in the resulting image were “unmixed” by the software, and a composite image was produced, allowing spectral characterization and quantitation for each of the multi-labeled components in an image while removing any autofluorescence. Immunofluorescent controls included the omission of the primary antibody from the immunostaining protocol and pre-adsorption with the respective antigens for each primary antibody, which completely eliminated staining for the corresponding antigen.

Slides were analyzed in ImageJ Software (National Institutes of Health, Bethesda, MD) to measure the length of each adrenal cortical layer. Layers were measured in microns, with the immunofluorescence and 4′,6-diamidino-2-phenylindole counterstain used to distinguish the morphological differences in each layer (*zona glomerulosa* (zG), *zona fasciculata* (zF), and *zona reticularis* (zR)).

### Aldosterone Analysis

Aldosterone was measured using an EIA kit (ENZO^®^ Life Sciences; ADI-900-173). The optical density of each sample was read at 405 nm using the Molecular Devices SpectraMax ID5. The concentration of aldosterone (pg/mL) was determined using the Softmax Pro microplate data software and presented as a ratio of cell protein level.

### RNA Extraction

RNA extraction was performed using the Qiagen (Valencia, CA) RNeasy Microarray Tissue kit (73304). Tissue was placed in QIAzol lysis reagent and homogenized for 30-40 seconds. Following homogenization, the homogenate was placed on the bench top at room temperature for 5 minutes to promote dissociation of the nucleoprotein complexes. Chloroform (200 µL) was added, mixed and allowed to sit at room temperature for 3 minutes. Homogenates were then centrifuged at 12,000 x g for 15 minutes at 4°C. The upper aqueous layer was removed and mixed with 600 μL of 70% ethanol. A RNA easy Mini spin column along with DNAse digestion set (Qiagen; 79254) were used for purification. Concentration (ng/μL) and purity (260/280 ratio) were determined using the Thermo Scientific (Pittsburgh, PA) NanoDrop ND-1000 Spectrophotometer. cDNA synthesis was performed using the Bio-Rad iScript cDNA synthesis kit (Hercules, CA; 170-88912).

### Real-Time PCR Analysis

PCR analysis was performed for angiotensin II type 1 receptor (AT_1_R), 11β- hydroxylase (CYP11B1), aldosterone synthase (CYP11B2), steroidogenic acute regulatory protein (StAR) and glyceraldyhyde 3-phosphate dehydrogenase (GAPDH) was used as the housekeeping gene. The DNA sequence for each gene was obtained from the National Center for Biotechnology Information (NCBI). The primer pair products were validated by gel electrophoresis and DNA sequence analysis to original gene (**Table 1**). PCR was carried out using the Bio-rad iCycler iQ real-time detection system with Bio-Rad iQ SYBR Green Supermix. One cycle was performed at 95°C for 10 minutes to activate polymerase and denature the DNA. Fluorescence data collection was performed for 40 cycles at 95°C for 15 seconds and 40 cycles at 58°C for 1 minute. The fold change of each target mRNA expression relative to GAPDH under experimental and control conditions were calculated based on the threshold cycle $({\text{C}}_{\text{T}})\text{ as}\text{r}=2^{-\Delta (\Delta {\text{C}}_{\text{T}})}$, where ΔC_T_ = C_T_(target)−C_T_(GAPDH) and Δ(ΔC_T_) = ΔC_T_(experimental)−ΔC_T_(control). The amplicons for all primer sets were sequenced and validated.

**Table 1:** Primer squences.

Gene	Forward primer (5’- 3’)	Reverse primer (5’- 3’)	Amplificate size (bp)	Reference Sequence
GAPDH	GGTGATGCTGGTGCTGAGTA	GGATGCAGGGATGATGTTCT	369	NM_017008.4
				
CYP11B1	TTACCCAAGAGCTTGACTCGTTGGAC	ACCATCTCGGATATGACACTCCAG	161	XM_039079756.1
				
CYP11B2	ACGAGGTAGCAAGGGACTTCTTGGA	GCATGGATGAACTTCAGGCTACCA	193	XM_039078395.1
				
StAR	AAGAAGGAAAGCCAGCAGGAGAATGGA	TGCGGTCCACCAGTTCTTCATAGAGTC	133	NM_031558.3
				
AT1R	GCTAAGCAGCTCACTCACTAC	AACTCTTGACCTCCCATCTC	363	NM_031009.2

GAPDH, Glyceraldehyde-3-Phosphate Dehydrogenase; CYP11B1, 11-beta hydroxylase; CYP11B2, aldosterone synthase; StAR, Steroidogenic acute regulatory protein;

AT1R, Angiotensin II receptor type 1.

### Protein Extraction and Quantification

Media was removed and saved for aldosterone analysis. Cells were rinsed with PBS and put on ice. While remaining on ice, 50 µL of RIPA buffer with Halt was added to each of the twelve wells and cells were harvested and homogenized while on ice. The protein content of samples was then determined by the Bio-Rad protein assay (Bradford and Lowry) by following the manufacturer recommendation (20).

### Statistical Analysis

One-way ANOVA were performed in experiments 1, and 2 for adrenal morphology and peak/AUC aldosterone plasma concentrations. A 2-way ANOVA followed by post-hoc Bonferroni paired-comparisons were performed in experiments 2, 3, 4 and 5 for aldosterone plasma concentrations over timed samples, peak/AUV aldosterone across days of treatment, culture media aldosterone concentrations across atrazine concentrations or duration and angiotensin II treatment, and blood pressure parameters. Cardiac mechanical parameters are presented as either baseline values (values before angiotensin II administration) or as a change from baseline (peak response to an angiotensin II). Multiple t tests were performed on the effect of each angiotensin II dose in vehicle and atrazine treated rats. Statistical analysis for the real-time PCR data was performed using a modification of the ΔCt (21). The level of statistical significance was set at *P* ≤ 0.05 for all statistical tests. All values were reported as the mean ± SEM. Prism 8 for Mac was used for all analysis (GraphPad Software, Inc., La Jolla, CA).

## Results

### Experiment 1: Effects of Atrazine or Restraint Stress Treatment on Adrenal Morphology

**Figure 1:**
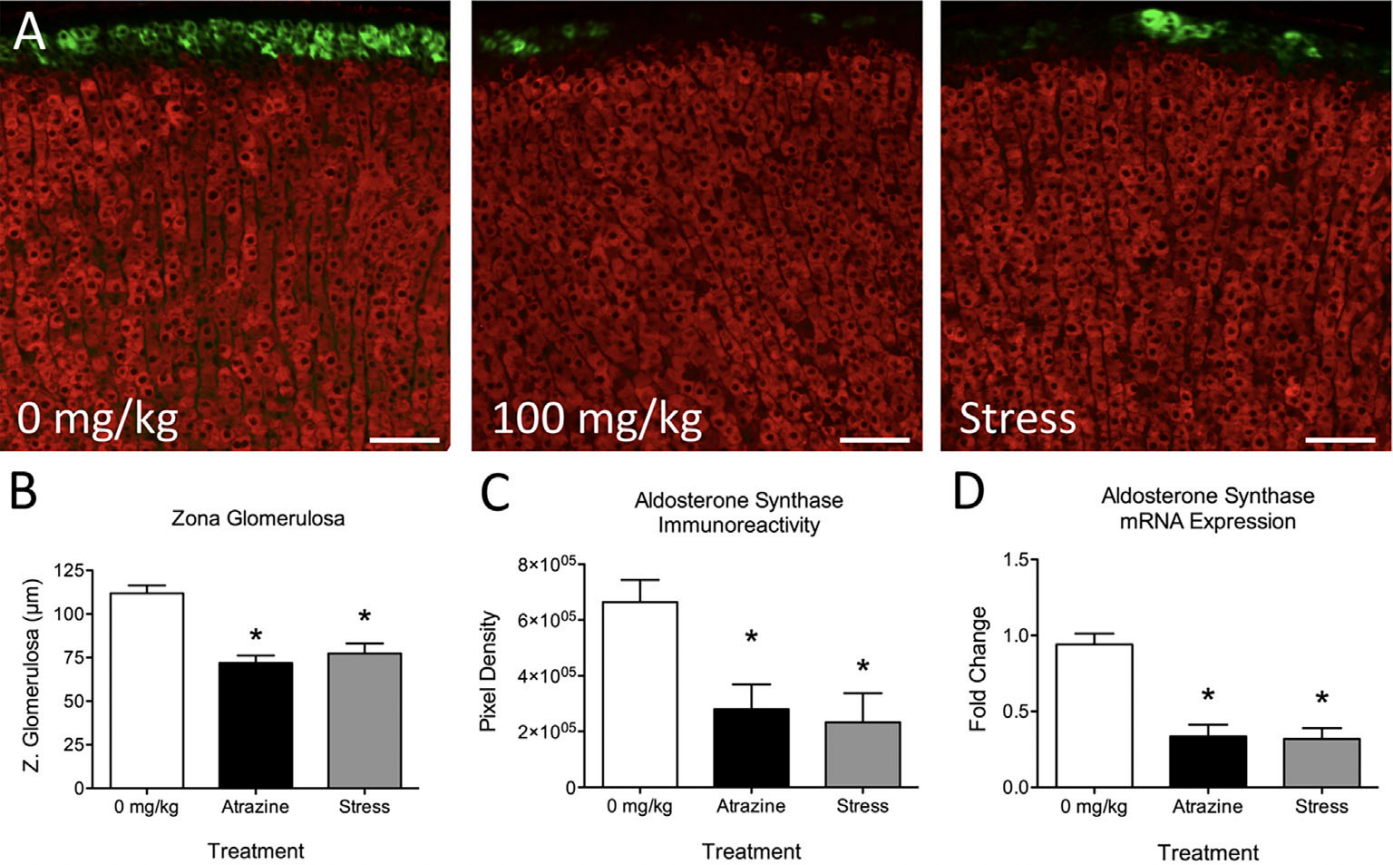
Atrazine treatment and repeated restraint stress reduce zona glomerulosa thickness and aldosterone synthase immunoreactivity and expression. **(A)** Representative images of the adrenal cortex of vehicle, atrazine (100 mg/kg), and restraint stressed animals displaying aldosterone synthase (green) and 11β-Hydroxylase immunofluorescence. **(B–D)** Histograms depicting **(B)** mean zona glomerulosa thickness, mean aldosterone synthase **(C)** immunoreactivity and **(D)** expression in adrenals of vehicle, atrazine (100mg/kg), and restraint stressed animals after 4 days of treatment. Data presented as the mean ± SEM. Asterisks signify difference from vehicle group (*P* < 0.05).

Atrazine treatment had no statistically significant effects on adrenal cortical or medulla thickness (**Table 2**; *F*_(2, 21)_ = 0.91, *P* = 0.91; *F*_(2, 21)_ = 0.136, *P* = 0.874, respectively). When adrenal cortical zones were analyzed, the zG was found to be reduced in thickness after 4 days of treatment with 100 mg/kg BW of atrazine or 30 min of restraint stress when compared with vehicle-treated animals (**Table 2**, **Figure 1A**; *F*_(2, 21)_ = 20.8, *P* < 0.001). Atrazine or restraint stress treatments had no effect on the thickness of the zF or zR (*F*_(2, 21)_ = 3.09, *P* = 0.066; *F*_(2, 21)_ = 0.85, *P* = 0.44, respectively). There was no treatment effect on cell density in the zG or zF (*F*_(2, 21)_ = 1.37, *P* = 0.277; *F*_(2, 21)_ = 2.09, *P* = 0.149). Cell density was lower in the zR of atrazine treated animals compared to vehicle treated animals (*F*_(2, 21)_ = 9.00, *P* < 0.005). There were no differences in adrenal weights (% BW) between treatment groups (*F*_(2, 21)_ = 0.437, *P* = 0.652). Along with decreased zG layer thickness (**Figure 1B**), atrazine and stressed animals had less immunoreactivity (**Figure 1C**, *F*_(2, 21)_ = 5.10, *P* = 0.016) and expression (**Figure 1D**, *F*_(2, 21)_ = 20.87, *P* < 0.001) for aldosterone synthase There was no significant effect of treatment on animal body weight (**Supplementary Table 1**, *F*_(2, 84)_ = 1.56, *P* = 0.217).

**Table 2:** Effects of atrazine or stress treatment on adrenal morphology.

Adrenal Metrics	Vehicle			Atrazine				Stress	
zG Thickness (μm)	111.90	4.60	8	71.74	3.76	8*	77.41	5.73	8*
zF Thickness (μm)	729.01	18.50	8	778.33	27.73	8	819.83	29.92	8
zR Thickness (μm)	477.87	28.51	8	346.74	14.53	8	433.24	28.81	8
Cortical Thickness (μm)	1318.78	49.04	8	1305.81	34.09	8	1330.48	32.75	8
Medulla (μm)	1022.77	67.22	8	1060.67	75.38	8	1013.63	59.80	8
zG Cell Density	40.23	0.99	8	43.77	1.06	8	42.77	1.22	8
zF Cell Density	12.66	0.59	8	11.63	0.40	8	12.93	0.41	8
zR Cell Density	27.27	0.85	8	22.53	0.88	8*	25.17	0.62	8
Adrenal Weight (% BW)	0.0152	0.00091	8	0.0156	0.00097	8	0.0164	0.00089	8

Data presented as the mean ± SEM. Asterisk signifies difference from control group (P < 0.05).

### Experiment 2: Effects of Atrazine or Restraint Stress on Angiotensin II Induced Aldosterone Release

**Figure 2:**
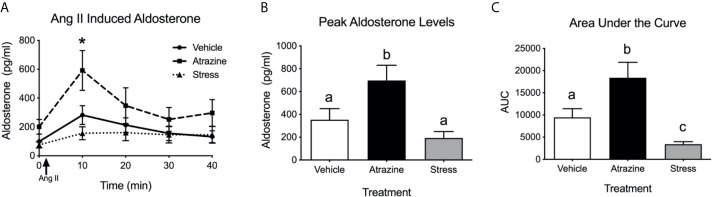
Atrazine treatment increases circulating aldosterone concentrations after angiotensin II (Ang II). **(A)** Line graph depicting aldosterone concentrations before and after infusion of Ang II in vehicle, atrazine (100mg/kg), and restraint stressed animals after 4 days of treatment. Arrow indicates timing of Ang II (5 µg/kg of BW). Histograms depicting **(B)** peak aldosterone concentrations and **(C)** area under the curve levels in vehicle, atrazine (100mg/kg), and restraint stressed animals after 4 days of treatment. Data presented as the mean ± SEM. Asterisk signifies difference from control group (*P* < 0.05). Letters denote significant difference between groups.

Intravenous infusion of angiotensin II resulted in higher plasma aldosterone levels in all treatment groups (**Figure 2A**, *F*_(1, 38)_ = 14.0, *P* = 0.0005). There was no significant difference in baseline aldosterone levels. At the 10 min timepoint, atrazine treated animals had a higher concentration of aldosterone compared to vehicle and stress treated groups (*F*_(8, 76)_ = 3.47, *P* = 0.0019). Atrazine treated animals displayed a higher peak plasma aldosterone concentration after Ang II treatment compared to vehicle and restraint stressed animals, independent of timepoint of peak aldosterone (**Figure 2B**; *F*_(2, 18)_ = 6.8, *P* = 0.0068). The stressed animals had a delayed time to peak compared to both vehicle and atrazine treated groups (vehicle=12.22 min, atrazine=10.0 min, stressed=21.67; *F*_(2, 18)_ = 5.1, *P* = 0.02). The area under the curve (AUC) levels were significantly different between all groups (**Figure 2C**; *F*_(2, 18)_ = 10.5, *P* = 0.0009). The mean AUC for atrazine treated animals was significantly higher than vehicle animals and stressed animals had significantly lower AUC levels compared to vehicle treated animals. There was no significant effect of treatment on animal body weight (**Supplementary Table 1**, *F*_(2, 72)_ = 0.88, *P* = 0.42).

### Experiment 3: Effects of Atrazine Treatment Duration on Angiotensin II Induced Aldosterone Release

**Figure 3:**
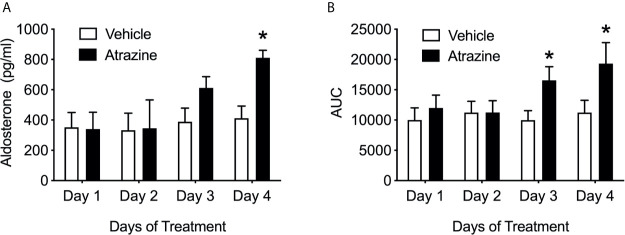
Atrazine treatment increases circulating aldosterone concentrations after angiotensin II over 4 days of treatment. Histograms depicting **(A)** peak aldosterone concentrations and **(B)** area under the curve levels in vehicle (white bars) and atrazine (100mg/kg, black bars) after 1, 2, 3, and 4 days of treatment. Data presented as the mean ± SEM. Asterisk signifies difference from control group (*P* < 0.05).

Only animals treated with atrazine for 4 days demonstrated a significantly increased peak Ang II-induced aldosterone (**Figure 3A**; *F*_(1, 40)_ = 4.2, *P* = 0.047). There were no significant differences in peak plasma aldosterone concentration between animals treated with atrazine or vehicle for 1, 2, or 3 days in their response to Ang II. Animals treated with atrazine for 3 or 4 days displayed significantly larger AUC compared to vehicle treated animals (**Figure 3B**; *F*_(1, 40)_ = 6.7, *P* = 0.013). There was no effect of atrazine treatment on AUC after 1 or 2 days of treatment. There was no significant effect of treatment on animal body weight (**Supplementary Table 1**, *F*_(1, 56)_ = 2.68, *P* = 0.11).

### Experiment 4: Effects of Atrazine Treatment on Aldosterone Production in Human Adrenocortical Cells

**Figure 4:**
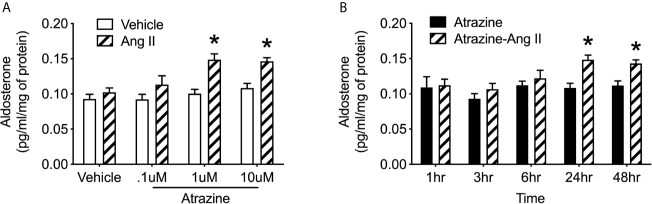
Atrazine increases Ang II-induced aldosterone synthesis in human adrenal cell lines. **(A)** Histogram depicting mean media concentration of aldosterone (pg/ml/mg) in cell cultures treated with atrazine (.1, 1.0, or 10.0 µm) or atrazine plus Ang II (0.1 nM). Atrazine treatment was 24 h in duration. **(B)** Histogram depicting mean media concentration of aldosterone (pg/ml/mg) in media of H295R cell cultures treated with atrazine (1 µm; solid black bars) or atrazine plus Ang II (0.1 nM). Atrazine treatment was 1, 3, 6, 24, or 48 h in duration. Data presented as the mean ± SEM. Asterisk signifies difference from control group (*P* < 0.05).

H295R cells were treated with 0.1, 1.0, or 10 µM atrazine for 24 hours then stimulated with Ang II (0.1 nM). Preliminary work demonstrated that 0.1 nM Ang II treatment of cultured H295R cells failed to elicit a significant increase in aldosterone concentrations in culture media (**Supplementary Figure 1**). This dose was used to investigate possible increased response to Ang II due to atrazine. Cultures pretreated with 1.0 or 10 µM of atrazine and then stimulated with Ang II had significantly elevated aldosterone concentrations compared to cultures pretreated with atrazine alone (**Figure 4A**; *F*_(1, 16)_ = 29.7, *P* < 0.0001). Vehicle or 0.1 µM atrazine treated cultures did not display increased aldosterone concentrations in the media after Ang II treatment. H295R cells were subsequently treated with 10 µM atrazine for 1, 3, 6, 24, or 48 hours then treated with Ang II. Cultures treated for 24 or 48 hours displayed a significant increase in media aldosterone concentration (**Figure 4B**; *F*_(1, 20)_ = 29.7, *P* < 0.0001).

### Experiment 5. Effects of Atrazine Treatment on Blood Pressure and Heart Rate

**Figure 5:**
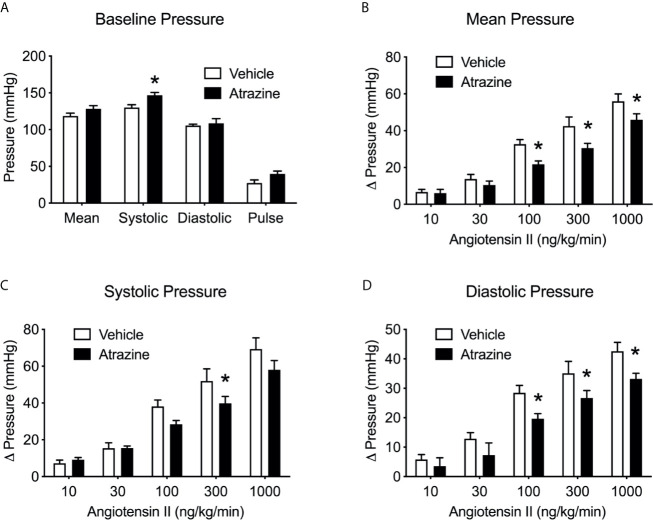
Atrazine treatment alters basal arterial pressure and attenuates pressor effects of Ang II infusion. Histograms depicting the baseline blood pressure **(A)** in vehicle and atrazine (100 mg/kg) treated animals and changes in mean **(B)**, systolic **(C)** and diastolic **(D)** pressures in vehicle or atrazine treated animals during angiotensin infusion (10-1000 ng/kg/min). Data presented as the mean ± SEM. Asterisk signifies difference from control group (*P* < 0.05).

Atrazine treatment increased basal systolic arterial pressure in anesthetized rats but had no effect on diastolic arterial pressure, mean arterial pressure, pulse pressure or heart rate. (**Figure 5A**, t(8) = 3.1, *P* = 0.015). Ang II infusion caused a dose dependent increase in arterial pressure but atrazine treated animals displayed lower responses to Ang II infusions compared to vehicle treated control animals (**Figure 5B**, *F*_(1, 37)_ = 15.3, *P* = 0.0004), systolic (**Figure 5C**, *F*_(1, 37)_ = 6.03, *P* = 0.019), and diastolic pressure (**Figure 5D**, *F*_(1, 37)_ = 14.4, *P* = 0.0005). There was no significant effect of treatment on animal body weight (**Supplementary Table 1**, *F*_(1, 32)_ = 0.53, *P* = 0.47).

### Experiment 6. Effects of Atrazine Treatment on Select Gene Expression

**Figure 6:**
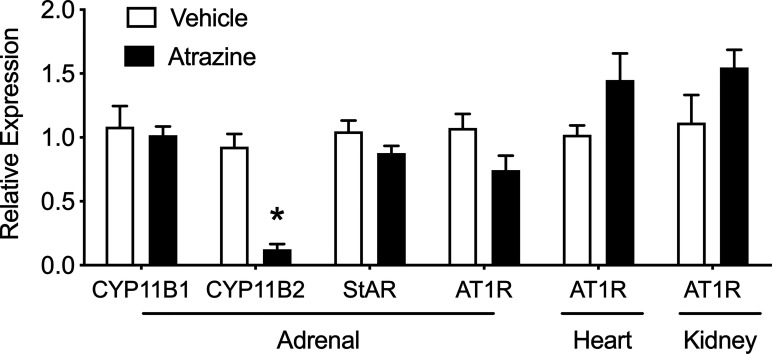
Effects of atrazine treatment on select gene expression of the adrenal, heart and kidney. Histogram depicting the fold change expression of CYP11B1 (11β-Hydroxylase), CYP11B2 (aldosterone synthase), steroidogenic acute regulatory (StAR) protein, angiotensin II receptor type 1 (AT_1_R) expression in the adrenal, heart, and kidney of vehicle (clear bar) or atrazine (black bar, 100 mg/kg). Data presented as the mean ± SEM. Asterisk signifies difference from control group (*P* < 0.05).

There was no effect of atrazine treatment on the adrenal expression of CYP11B1, StAR, and AT1R (**Figure 6**, t(8) = 0.32, *P* = 0.76; t(8) = 1.65, *P* = 0.138; t(8) = 0.32, *P* = 0.76; t(8) = 0.2.1, *P* = 0.069). However, aldosterone synthase (CYP11B2) was significantly lower in atrazine treated animals compared to controls (t(8) = 7.43, *P* < 0.0001). In heart and kidney tissues, there was no effect of atrazine treatment on AT1R expression (t(8) = 1.93, *P* = 0.088; t(8) = 1.69, *P* = 0.13, respectively). There was no significant effect of treatment on animal body weight (**Supplementary Table 1**, *F*_(1, 32)_ = 0.70, *P* = 0.41).

## Discussion

We and others have demonstrated that atrazine acts centrally to induce a “stress-like” response resulting in a rapid rise in ACTH and corticosterone plasma concentrations (11, 13, 16, 22, 23). Prolonged atrazine treatment can lead to altered adrenal and thymus weights (24–26) which are also identified in repeatedly stressed animals with sustained elevated stress hormones (27–30). Specifically, we have demonstrated that repeated atrazine exposure can alter adrenal cortical morphology with a reduction in zG thickness (11). At the time of previous reports, we attributed atrazine-induced adrenal morphology changes to repeated adrenal stimulation *via* ACTH release because the morphology change mimicked those of repeatedly stressed animals and atrazine had no direct, stimulatory effects on the adrenal (11, 14, 31). In the current work, we tested this theory by treating animals with atrazine, vehicle, or restraint stress for 4 days (**Figure 1**, **Table 2**). There was a significant reduction in aldosterone synthase immunoreactivity and expression in both atrazine and stressed groups when compared to control animals. Similarly, there was a significant reduction in the zG thickness in both stressed and atrazine treated groups compared to control animals. Chronically stressed animals present with hyperplasia and hypertrophy in the zF, and reduced cell size in the zona glomerulosa (14). The 4-day treatment duration in the present study did not result in significant zF changes but did reduce zG thickness. The present findings suggest activation of the stress response or ACTH treatment first acts to reduce zG activity then increases zF thickness over a longer time period (32). There was no difference found between stressed and atrazine treated animals except for a reduced cell density in the zF. There was a significant reduction in zona glomerulosa thickness in atrazine and stressed treated animals with no change in other cortical layers or total cortical thickness. The lack of significant change in total cortical thickness is explained by a matter of proportion. The adrenal cortex is approximately 90% zona fasciculata and zona reticularis combined. Therefore, small but significant changes to the zona glomerulosa thickness is insignificant when combined with other cortical zones.

The acute rate-limiting step mediating aldosterone production includes the activation of steroidogenic acute regulatory protein (StAR). StAR regulates the transport of cholesterol from the outer to the inner mitochondrial membrane. The transcriptional and translational regulation of StAR controls the expression level of the 37 kDa StAR protein (33–37). Aldosterone synthase (CYP11B2) present in the zG catalyzes the final steps in the production of aldosterone from cholesterol acetate. This is accomplished in three separate reactions converting 11-deoxycorticosterone to aldosterone *via* intermediates corticosterone and 18-hydroxycorticosterone by aldosterone synthase (38). ACTH in repeatedly stressed animals or cultured zG cells causes regression of the zona glomerulosa and suppression of aldosterone synthase expression (14, 39, 40). Similarly, low-dose infusion of ACTH in human subjects results in an initial increase in plasma aldosterone levels during the first 12–36 h, but a slow decline in these values in subsequent days (41). The mechanism for ACTH-mediated repression of aldosterone production and aldosterone synthase expression remains unknown. One theory postulates that repeated ACTH stimulation induces CYP11B1 and CYP17, the activities of which direct the precursors of the steroidogenic pathway away from the production of aldosterone, and towards that of cortisol or corticosterone (42). CYP11B2 immunoreactivity and expression were significantly lower in atrazine and stressed animals (**Figure 1**).

To determine if the reduction in aldosterone synthase in the zG of stressed and atrazine treated animals would lead to reduced adrenal response to stimulation, animals were restraint stressed or treated with atrazine for 4 days and then challenged with Ang II. As predicted, stressed animals, after Ang II stimulation, had lower AUC aldosterone plasma concentrations when compared to vehicle and atrazine treated groups (**Figure 2**). While this is the first *in vivo* report, attenuated Ang II-induced aldosterone release from adrenal tissue from repeatedly restraint stressed animals has been reported previously (31). In contrast to stressed animals, after Ang II stimulation, atrazine treated animals had higher circulating aldosterone concentrations in both peak levels and AUC when compared to vehicle and stressed animals. This finding led us to reject our original hypothesis, that repeated stress and atrazine treatment would have similar effects on the zG resulting in lowered aldosterone secretion after stimulation.

To better understand the surprising findings of increased aldosterone release in atrazine treated animals, the response to Ang II stimulation was assayed after 1, 2, 3, or 4 days of atrazine treatment. Plasma atrazine concentrations peak 2 hours post gavage in the rat with the majority of atrazine and its metabolites cleared from the animal by 24 hours (43, 44). If atrazine potentiated aldosterone producing cells in a rapid pharmacological manner, aldosterone concentrations would predictably be as high or higher in 1 or 2 day treated groups than animals treated for 4 days because atrazine-induced ACTH would not have driven down aldosterone synthase expression after 1 or 2 exposures. However, there was no difference in aldosterone concentrations between atrazine and vehicle treated groups after 1 or 2 days of treatment (**Figure 3**). After 3 and 4 days of atrazine treatment, the AUC of plasma atrazine concentrations were higher than vehicle treated groups. Peak aldosterone concentrations after Ang II treatment were only significantly above control animals after 4 days of atrazine treatment suggesting the need for multiple exposures and cell physiological changes, most likely, dependent on gene expression changes. While not definitive, our current findings suggest that the increased aldosterone production in atrazine treated animals is not due to a short-term pharmacological action of atrazine but more likely at the level of gene regulation, particularly at the levels of AT1 receptor, StAR or CYP11B2. However, while CYP11B2 was reduced, atrazine treatment had no effect on adrenal AT1 receptor or StAR expression (**Figure 6**).

Aldosterone production is primarily regulated by the renin-angiotensin-aldosterone system which involves multiple organs and organ systems including the kidneys, gastrointestinal tract, and ingestive behavior. To reduce these variables, human adrenocortical carcinoma cells, H295R, were pretreated with atrazine. A dose response experiment identified an Ang II concentration to minimally stimulate aldosterone release in culture. This allowed for the identification of possible atrazine-induced potentiated stimulation (**Supplemental Figure 1**). Adrenal cells pre-treated with 1 or 10 µM of atrazine followed by Ang II stimulation had higher aldosterone concentrations in their media (**Figure 4A**). However, aldosterone concentrations were only elevated in atrazine pre-treated cultures plus Ang II after 24 and 48 hours but not after 1, 3, or 6 hours of atrazine treatment (**Figure 4B**). Similar to *in vivo* findings, atrazine induced-aldosterone potentiation *in vitro* does not occur when extracellular atrazine concentrations are at their highest but after prolonged exposure. This further supports atrazine acting through gene alteration rather than acute pharmacological effects.

Atrazine treated animals presented with significantly higher systolic baseline blood pressure and a significantly reduced response to Ang II in arterial, systolic, and diastolic pressure (**Figure 5**). Atrazine treatment has previously been linked to cardiovascular issues in mice (45) and dogs (46). Treatment with atrazine or its metabolite diaminochlorotriazine (DACT) to male and female beagles results in significant signs of cardiovascular issues including cardiac lesions, cardiac hypertrophy, and atrial fibrillation (46–48). Similarly, male mice treated with atrazine present with cardiac lesions after 21 days of treatment (46). Although the reported cardiac issues associated with atrazine treatment are vague and not well characterized, prolonged elevated plasma aldosterone concentrations or hyper-aldosteronism resulting in elevated blood pressure can lead to atrial fibrillation, cardiac lesions, and cardiac hypertrophy (49–52).

Atrazine animals had reduced changes in blood pressure in response to Ang II. There was no difference in AT1R expression in heart tissue or kidney tissue (**Figure 6**). The mechanism of reduced response to Ang II stimulated blood pressure in atrazine treated animals is unknown. Perhaps atrazine animals are responding normally to Ang II stimulation but their elevated basal pressure results in a significantly smaller change from basal blood pressure. Atrazine treatment had no effect on blood pH or electrolyte levels suggesting no major alteration of kidney function (**Supplemental Table S2**). However, blood analysis was performed on atrazine and control animals not challenged with Ang II.

While all of our current evidence implicates a direct action of atrazine on aldosterone-producing cells of the adrenal, there are multiple other mediators of the renin-angiotensin-aldosterone system (RAAS) which need to be investigated further such as renin, angiotensin converting enzyme, and endogenous angiotensin concentrations. There may also be an autonomic response to atrazine exposure which could potentiate aldosterone release. The splanchnic innervation of the adrenal acts as an extra-angiotensin mechanism in the control of aldosterone secretion. Splanchnic nerve activation leads to release of catecholamines (epinephrine, norepinephrine, and dopamine). Catecholamines have been shown to directly stimulate aldosterone production (53). Ang II infusion can also potentiate the release of catecholamines from the adrenal medulla during splanchnic nerve stimulation (54). Atrazine could work to potentiate the paracrine stimulatory action of catecholamines on aldosterone release. However, elevated catecholamines are immunosuppressive and our work has demonstrated no immune toxicity in response to similar atrazine treatment (26, 55).

Our original report of atrazine-induced changes in adrenal morphology was performed in ovarectomized females to control for variation in the ovarian cycle and in conjunction to examining atrazine’s effects on luteinizing hormone pulsatile and pre-ovulatory surge release. Estrogens elicit direct vasodilatory effects *in vivo*, albeit they may affect blood pressure also through other mechanisms, including inhibition of sympathetic tone (56). In adrenal cells, estradiol, working through ERβ, inhibits CYP11B2 expression (57–59). The use of ovarectomized animals most likely increased the potential synthesis of aldosterone. Preliminary data suggests atrazine potentiation of Ang II-stimulated aldosterone release persists in estradiol treated animals. However, as predicted, treatment with estradiol reduces Ang II-induced aldosterone concentrations equally across treatments (data not shown). It may be more difficult to identify atrazine-induced reduction in zona glomerulosa thickness in intact females. Estrogen reduces zona glomerulosa activity and presumably cell size (60). Males should also be included in future investigations since males present with higher aldosterone levels than females and perhaps are more responsive to endogenous Ang II stimulation (61).

In the current study we have shown that adrenal cells of atrazine treated animals respond to Ang II with increased release of aldosterone compared with stressed or control animals. In addition, atrazine treatment results in enhancement of aldosterone release from cultured human adrenocortical cells. In addition, atrazine treated animals present with higher baseline blood pressure and possessed a lower response to Ang II treatment than vehicle treated control groups. Aldosterone plays a central role in the regulation of systemic arterial pressure through its systemic synthesis *via* the renin-angiotensin aldosterone cascade (62).

Pathological consequences of excess mineralocorticoid activity included high blood pressure and evidence of myocardial necrosis and fibrosis (63, 64). Recent work suggests aldosterone has widespread cardiovascular and metabolic effects, beyond its effects on fluid and electrolyte balance (65). Aldosterone increases superoxide generation (66, 67). This may involve decreases in glucose-6-phosphate dehydrogenase activity and increases in NADPH oxidase. Atrazine treatment has also been linked to increased oxidative stress but the mechanism is not known (21, 68, 69). Atrazine treated animals displayed increased Ang II-induced aldosterone plasma concentrations after 3 and 4 days of treatment but not after 1 or 2 days of atrazine treatment. After 4 days of atrazine treatment, blood pressure was elevated. The required duration of atrazine dosing suggests possible prolonged effects. Roger et al. (2014) reported that rat dams treated with 125 mg/kg of atrazine over gestational days 16 through 20 produced adult offspring with elevated basal blood pressure (70). It is not known how long the atrazine-induced increased aldosterone stimulation will be sustained after treatment termination but the long-term effects on blood pressure after gestational exposure may suggest similar mechanisms at play.

As stated previously, aldosterone synthesis is primarily regulated by the rate limiting enzymatic work of StAR and CYP11B2. While expression of StAR and CYP11B2 levels are correlated with the degree of aldosterone synthesis, there are post-translational modification and phosphorylation events that can mediate their activity. Translational regulation controls the level of expression of the 37 kDa StAR protein, while phosphorylation of StAR is crucial for its activity (33–37). Increased intracellular calcium levels, following Ang II treatment, causes increased StAR activation in the H295R cell line (71–73) as well as primary cultures of bovine adrenocortical cells (74, 75). Atrazine has been shown in multiple cell types to be a specific phosphodiesterase (PDE) inhibitor (76–80). In adrenal cells, inhibition of PDE activity is associated with increased steroidogenesis (81, 82). Some Cushing syndrome cases have been linked to mutations to PDE subtypes (83). Although atrazine treatment does not directly stimulate zG cells, it may inhibit PDE activity and thus allow enhanced stimulation of zG cells in response to Ang II treatment. However, the timing and duration of atrazine treatment (>2 days) required for increased Ang II response does not support a direct pharmacological mechanism on PDE, however, additional work is needed to fully investigate a role for PDE inhibition in atrazine’s effects on aldosterone synthesis.

Due to its convenience of use, low cost, weed control efficacy, and relatively long half-life in the environment, atrazine is one of the most commonly identified herbicides in water sources (84, 85). While banned in many countries, atrazine is widely used in the United States and is often found in water sources above the Environmental Protection Agency’s mandated limit (4, 86, 87). Atrazine exposure in humans and animals has been linked to cardiovascular changes with no defined underlining cause (45, 70, 88–91). Future investigations will focus on the long-term effects of atrazine exposure on aldosterone regulation and establishing a No Observed Adverse Effect Level (NOAEL). Indeed, more work is needed to understand the effects of atrazine on aldosterone production and the ramifications of atrazine exposure to the renin-angiotensin-aldosterone system, blood pressure, kidney function, and the possible long-term effects of their disruption.

## Data Availability Statement

The raw data supporting the conclusions of this article will be made available by the authors, without undue reservation.

## Ethics Statement

The animal study was reviewed and approved by Animal Care and Use Committee of Auburn University.

## Author Contributions

CF, LM, and AZ contributed to conception and design of the study. DS, CR, RK, and MJ were instrumental in performing experiments. CF and LM performed the statistical analysis. CF and AZ wrote the first draft of the manuscript. All authors contributed to the article and approved the submitted version.

## Funding

This research was funded by the Animal Health and Disease Research Program, College of Veterinary Medicine, Auburn University.

## Conflict of Interest

The authors declare that the research was conducted in the absence of any commercial or financial relationships that could be construed as a potential conflict of interest.
