# Supplementary figures and images for: The Herbicide Atrazine Potentiates Angiotensin II-Induced Aldosterone Synthesis and Release From Adrenal Cells
Source: Front Endocrinol (Lausanne). 2021 Jul 14;12:697505. doi: 10.3389/fendo.2021.697505 (PMC8317615; doi:10.3389/fendo.2021.697505)

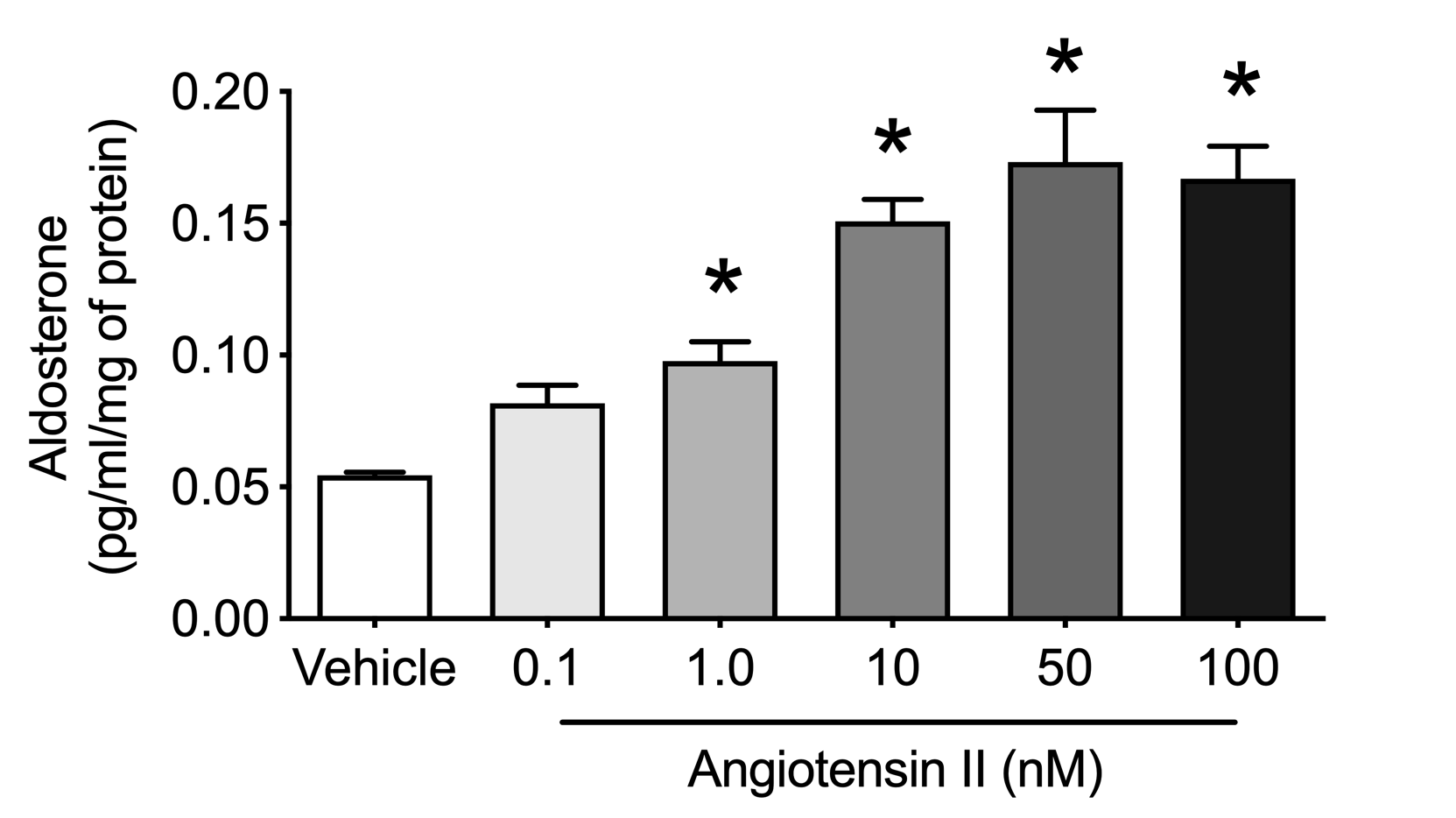

Supplement: Supplementary file 1 [file Image_1.tiff]

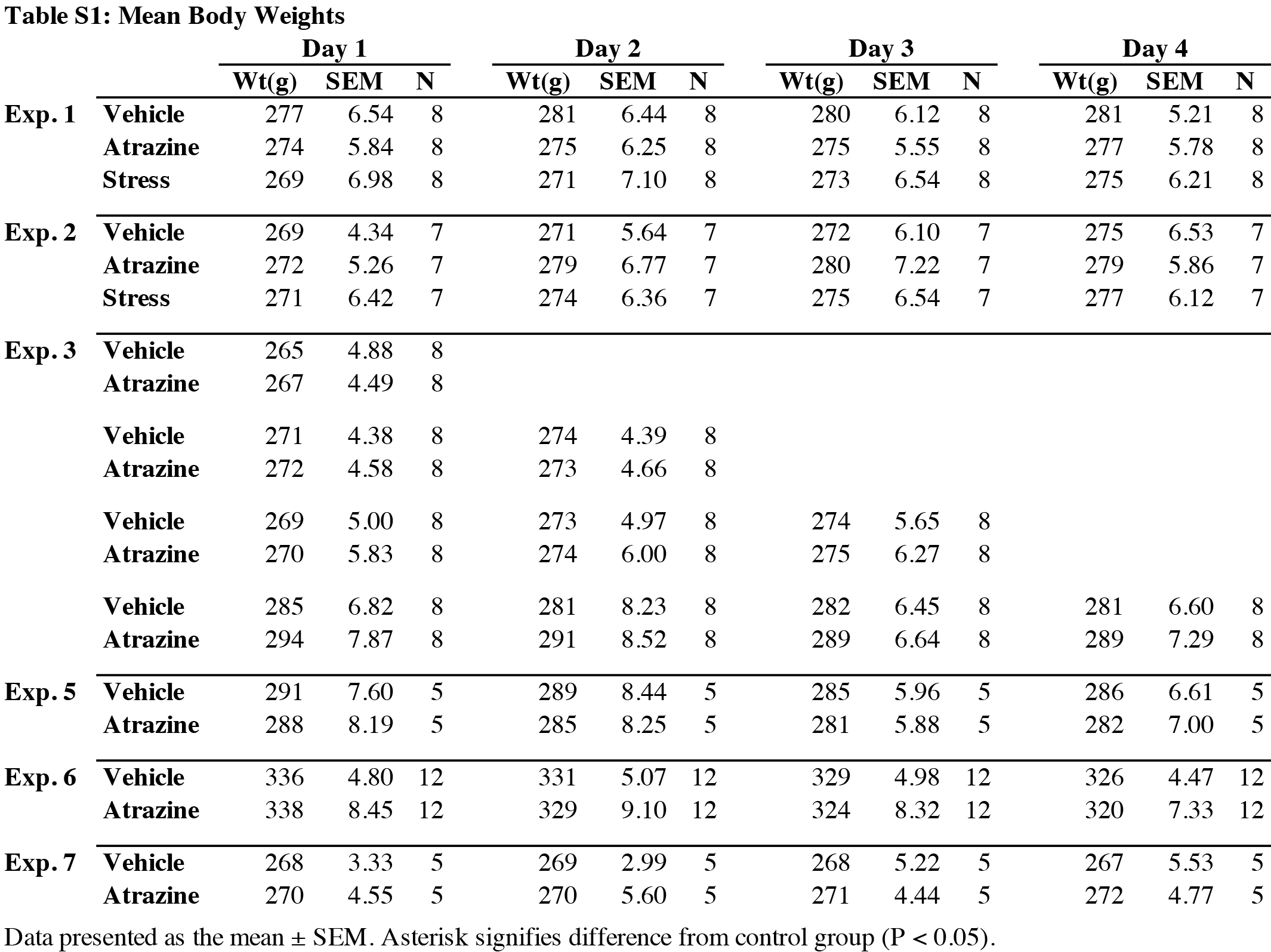

Supplement: Supplementary file 2 [file Image_2.tif]

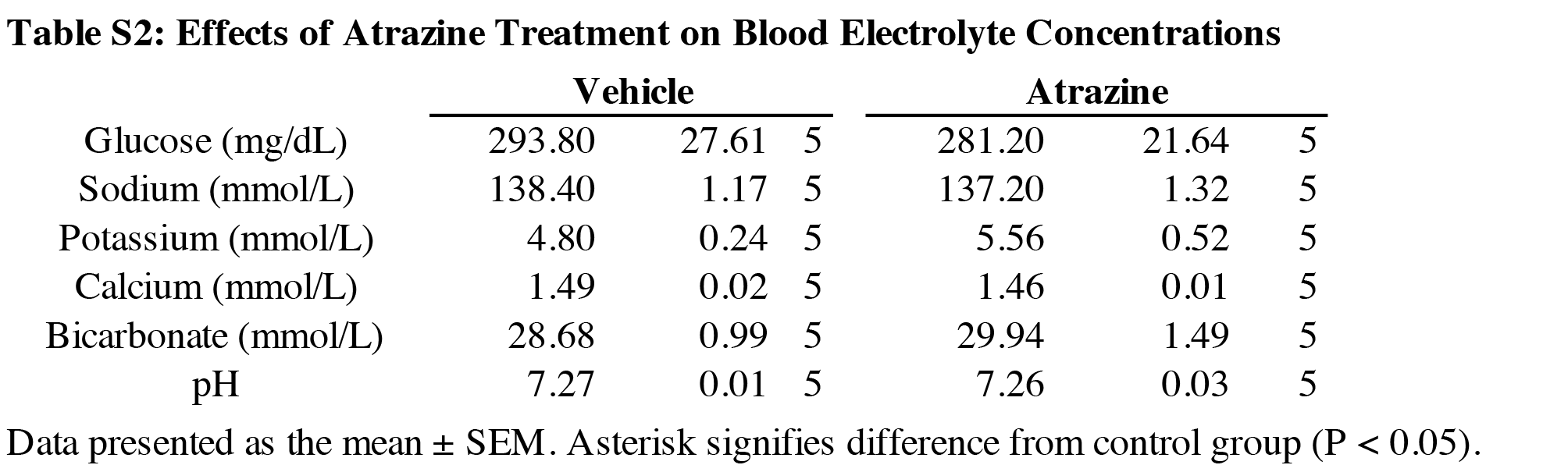

Supplement: Supplementary file 3 [file Image_3.tif]

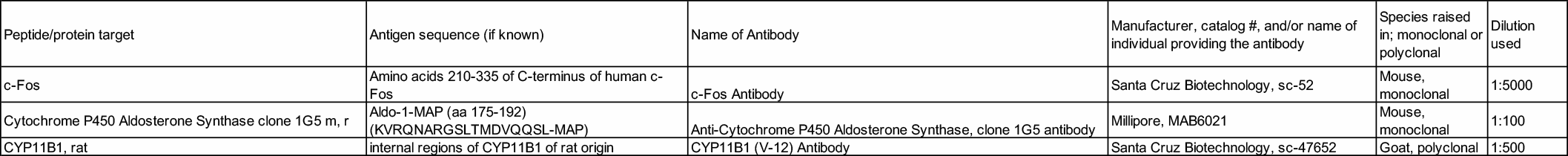

Supplement: Supplementary file 4 [file Image_4.tif]
